# Association Between Cardiorespiratory Fitness and a Composite Index of Cardiac Autonomic Control: Potential Implications for Exercise Training Evaluation

**DOI:** 10.3390/jcm15176929

**Published:** 2026-09-07

**Authors:** Gianluigi Oggionni, Giuseppina Bernardelli, Mara Malacarne, Massimo Pagani, Antonio Pelliccia, Daniela Lucini

**Affiliations:** 1 BIOMETRA Department, University of Milan, 20129 Milan, Italy; gianluigi.oggionni@unimi.it (G.O.); mara.malacarne@unimi.it (M.M.); 2 Exercise Medicine Unit, IRCCS, Istituto Auxologico Italiano, 20135 Milan, Italy; g.bernardelli@unimi.it (G.B.); m.pagani@auxologico.it (M.P.); 3 DISCCO Department, University of Milan, 20122 Milan, Italy; 4 Department of Medicine, Institute of Sport Medicine and Science, 00197 Rome, Italy; antonio.pelliccia@coni.it

**Keywords:** cardiopulmonary exercise testing, exercise medicine, heart rate variability, autonomic nervous system, cardiorespiratory fitness, preventive cardiology

## Abstract

**Background/Objectives**: Cardiorespiratory fitness (CRF), measured as VO_2_peak during cardiopulmonary exercise testing (CPX), represents a fundamental marker of cardiovascular health. Heart Rate Variability (HRV) assesses cardiac autonomic regulation (CAR) non-invasively, though technical issues limit clinical use. Combining CRF and CAR may provide complementary information on cardiovascular and autonomic function. We examined the relationship between CRF and HRV using a composite autonomic index, the Autonomic Nervous System Index (ANSI), which incorporates multiple HRV variables accounting for age/sex-related influences. **Methods**: This cross-sectional study involved 200 adults undergoing preventive health evaluation without overt cardiometabolic disease. CRF was expressed as VO_2_peak and as VO_2_peak percentile from the FRIEND registry. CAR was assessed at rest using HRV. **Results**: We observed significant correlations between VO_2_peak and several HRV variables, with ANSI showing the strongest association (r = 0.457, *p* < 0.001). In simple regressions, ANSI was linked to VO_2_peak (R^2^ = 0.175, *p* < 0.001) and VO_2_peak percentile (R^2^ = 0.209, *p* < 0.001). In multiple regression, several autonomic variables were retained for VO_2_peak, whereas ANSI was the only autonomic variable retained for VO_2_peak percentile. This association remained significant after adjustment for age, sex, smoking, and anthropometric measures. Trend analyses (*p* < 0.001) and ordinal regression analyses (*p* < 0.001) further substantiated these findings. A 10-point increase in ANSI corresponded to an OR of 1.39 for belonging to a higher CRF percentile category. **Conclusions**: Multiple HRV indices were associated with CRF, with ANSI showing the strongest association. These findings highlight the potential of ANSI as a simple, integrated measure of CAR in individuals with different levels of CRF.

## 1. Introduction

Exercise training is widely considered a pivotal strategy for fostering well-being and preventing/treating many chronic diseases, ranging from cardiometabolic conditions to cancer [1,2,3,4,5]. It is strongly recommended by major prevention guidelines [1,2,5] and is expected to be prescribed by all physicians [3]. In particular, aerobic training is the primary tool for improving reduced cardiorespiratory fitness (CRF), which is a strong, independent predictor of mortality [1,2,5,6,7]. Nevertheless, a training program must be prescribed, taking into account specific clinical goals and patients’ clinical characteristics [8,9,10,11,12] to positively affect the pathogenic mechanisms underlying the disease.

Cardiopulmonary exercise testing (CPX) represents the state-of-the-art methodology for assessing CRF [2,7] and, hence, for monitoring the effects of aerobic exercise training [9,10,13] while also playing a pivotal role in exercise prescription by helping to define the appropriate training heart rate [9,10,11,13]. It constitutes an essential instrument for assessing the efficacy of aerobic training protocols and, ideally, should be considered a mandatory test for all healthy individuals or patients engaged in lifestyle modification programs based on aerobic exercise [2,14,15].

Several mechanisms may explain the association between reduced CRF, unhealthy lifestyles, and increased health risk, ranging from genetic factors to alterations in immunological and metabolic control and to dysregulation of the autonomic nervous system (ANS) [2,5,16,17,18,19,20]. Specifically, the importance of this regulatory mechanism is increasingly recognised, as autonomic dysregulation is a relevant pathway associated with many chronic conditions, such as ischaemic heart disease, hypertension, diabetes, obesity, cancer and stress syndrome [18,21,22]. Notably, dysregulation may manifest before the appearance of detectable clinical disease; for instance, it is characteristic of overweight states [18,23] or of arterial blood pressure within the high–normal range [24]. Conversely, the ANS is positively affected by aerobic exercise, improvements in body composition, smoking cessation, and stress management [23,25,26,27], suggesting a potentially convenient mechanism worth investigating to understand the physiological impact of exercise training and lifestyle interventions [28,29].

Although highly informative, the ANS remains challenging to study due to the complexity of analytical techniques and the difficulty of interpretation, particularly given the variety of methods used to assess it, the large number of autonomic indices derived, and the well-known influence of age and sex on cardiac autonomic control [30,31]. These factors often complicate interpretation and wider clinical application of HRV.

To overcome these difficulties, given the fundamentally unitary nature of visceral neural regulation, we previously introduced an integrated, composite Autonomic Nervous System Index (ANSI) [32,33,34]. ANSI integrates the information spread across multiple autonomic variables into a single composite measure. Its potential added value over individual HRV indices lies in providing a clearer appreciation of overall autonomic performance while reducing the complexity and potential redundancy associated with interpreting multiple autonomic indices [33].

Although ANS assessment using HRV is becoming increasingly popular [35,36,37,38,39,40], particularly in the sport and exercise fields, the important relationship between CRF and ANS control has mostly been considered in terms of improvements in ANS regulation following aerobic training; few studies have specifically examined its relationship with direct measurements of CRF using CPX. Previous research has often relied on indirect estimates of physical fitness, measurements from wearable devices, or small sample sizes, thereby limiting the robustness and physiological interpretability of their conclusions [41,42,43,44,45,46]. However, the relationship between ANSI and directly measured CRF, in comparison with individual HRV indices, has not yet been investigated.

Therefore, the aim of the present study was to assess the cross-sectional association of ANSI and individual HRV variables with VO_2_peak directly measured by CPX and with age- and sex-standardized FRIEND VO_2_peak percentiles in adults with varying levels of physical conditioning. We hypothesized that ANSI would be positively associated with directly measured CRF and would show a stronger association with CRF than individual conventional HRV indices.

## 2. Materials and Methods

### 2.1. Study Design and Participants

This observational, cross-sectional study was based on an analysis of data collected within an ongoing research program on autonomic nervous system assessment initiated in 2010. The study population comprised all eligible consecutive participants available in the database who fulfilled the inclusion and exclusion criteria. Eligible participants were adults aged ≥18 years who were able to complete a maximal CPX (Cardioline Cubeecg, Cardioline S.p.A., Milan italy; VmaxEncore, Viasys, Cardusion, San Diego, CA, USA) and had undergone a complete autonomic assessment. We excluded subjects with major cardiometabolic diseases (ischemic heart disease, heart failure, arrhythmias, diabetes, psychiatric diseases, oncological diseases, chronic pulmonary diseases, active orthopaedic conditions), those receiving pharmacological treatment with drugs that interfere with the autonomic nervous system (such as beta-blockers, dihydropyridine calcium channel blockers, or any antiarrhythmic agent), and professional athletes. The final study sample comprised 200 subjects (97 males, 48.5%; 103 females, 51.5%). All participants gave their written informed consent. The protocol of this study followed the principles of the Declaration of Helsinki and Title 45, US Code of Federal Regulations, Part 46, Protection of Human Subjects, Revised 13 November 2001, effective 13 December 2001 and was approved by the local Institutional Ethics Committee (Ethics Committee Istituto Auxologico Italiano, IRCCS, code 2022_01_25_03 dated 28 January 2022; and Ethics Committee Ospedale L.Sacco, Prot 651/09/70/AP, 23.12.09).

### 2.2. Clinical and Metabolic Assessment

All participants underwent a clinical assessment that included medical history, weight, height, waist circumference (WC), body mass index (BMI) calculation and haemodynamic parameters. In a subgroup of 89 subjects, fasting venous blood samples were collected to assess glucose levels and lipid profiles, when clinically indicated according to the individual cardiovascular risk profile.

### 2.3. Autonomic Assessment

Cardiac Autonomic Regulation (CAR) was assessed non-invasively using autoregressive spectral analysis of heart rate variability (HRV), as recently summarized [24,31,33,34]. Briefly, an electrocardiogram and respiratory activity (monitored with a piezoelectric belt from Marazza, Italy) were recorded on a computer. Beat-by-beat data analysis comprised a 5-min resting period followed by a 5-min upright recording, processed offline using HeartScope software (version 2.0). The program can analyze signals in both the time and frequency domains. To simplify the clinical interpretation of multiple HRV variables, we implemented a unified autonomic index, the Autonomic Nervous System Index (ANSI) [33,34], serving as a proxy for cardiac autonomic evaluation. As previously described, ANSI combines the three most informative autonomic indices: the RR interval (RR), the total power of RR interval variability (RR TP), and the difference between upright and resting low-frequency RR power expressed in normalized units (ΔLF) [33]. ANSI, by design free of age- and sex-related bias, is expressed as a percentile rank (0–100) and serves as a composite proxy for cardiac autonomic regulation, with higher values representing better autonomic function [33].

### 2.4. Cardiorespiratory Fitness Assessment

CRF was defined as the highest 10-s averaged value of oxygen consumption (VO_2_peak) during a standardized incremental cycle ergometer CPX with a target duration of about 10 (±2) min, with a respiratory quotient (RQ) ≥ 1.1 used as a criterion for maximal effort [7,10,47]. Expired air was collected using a breathing apparatus and analyzed with a metabolic measurement cart (VmaxEncore, Viasys, Carefusion, San Diego, CA, USA) to determine ventilatory and gas exchange variables on a breath-by-breath basis [7,10,47]. Given the well-known age- and sex-related influences on VO_2_peak, we used age-specific percentiles for males and females, based on recently updated reference standards for cycle-ergometer exercise testing [47].

### 2.5. Statistical Methods

Statistical analyses were conducted using IBM SPSS Statistics, version 29 (IBM Corp., Armonk, NY, USA). An a priori sample size calculation was performed for the planned regression analyses. Assuming the regression model would explain 10% of the variance in the outcome (R^2^ = 0.10), with α = 0.05, 80% power, and nine predictors, the minimum required sample size was deemed to be 150 participants; the final sample of 200 participants therefore exceeded this requirement. Continuous variables are presented as mean ± standard deviation (SD) for descriptive purposes. Between-sex comparisons were performed using the Mann–Whitney U test. All tests were two-tailed, and *p* < 0.05 was considered statistically significant. Pearson correlations were used to evaluate associations between cardiorespiratory fitness (CRF) and autonomic variables. Simple linear regression analyses were initially performed to assess the association of each autonomic variable with CRF. Multiple linear regression analyses were subsequently performed to evaluate the autonomic variables within the same analytical framework. Autonomic variables were entered using a forward-selection procedure based on the probability-of-F criterion (PIN = 0.05; POUT = 0.10). Standardized regression coefficients (β) were reported for variables retained in the models. To address potential confounding in the analyses of VO_2_peak percentile, two complementary adjusted models were performed. In the primary model, age, sex, smoking status, and body mass index (BMI) were entered a priori using the ENTER method, after which the autonomic variables were evaluated using the forward-selection procedure described above. In a parallel sensitivity analysis, BMI was replaced by waist circumference (WC), while age, sex, and smoking status were retained as the same a priori covariates. BMI and WC were not entered simultaneously because they were highly correlated and their inclusion together resulted in variance inflation factor (VIF) values > 5. Regression assumptions were evaluated before model interpretation by examining standardized residual distributions and normal probability plots, standardized residual-versus-predicted value plots, the Durbin–Watson statistic, tolerance values, and VIFs. Model explanatory power was quantified using the coefficient of determination (R^2^). Effect size was evaluated according to Cohen’s guidelines [48]. For categorical analyses, VO_2_peak percentile was additionally examined across nine predefined ordered percentile levels (10th through 90th percentiles), based on age- and sex-specific reference standards from the Fitness Registry and Importance of Exercise (FRIEND) [47]. Ordered trends across FRIEND percentile levels were assessed using the Jonckheere–Terpstra test with two-tailed Monte Carlo estimation. In addition, ordinal regression analysis using a proportional-odds model with a logit link was performed to examine the association between ANSI and ordered CRF percentiles. The proportional-odds assumption was assessed using the test of parallel lines [48].

## 3. Results

### 3.1. Characteristics of the Sample (Table 1)

Table 1 displays anthropometric, haemodynamic, haematochemical, autonomic, and cardiopulmonary exercise testing variables for the entire population and for males and females separately. Females and males showed significant differences in anthropometric variables, including weight, height and waist circumference (WC). Males showed higher systolic arterial pressure (SAP) (albeit within normal ranges), whereas diastolic arterial pressure (DAP) did not differ between sexes; resting heart rate (HR) also showed significant sex-related differences. Similarly, significant differences were observed in haematochemical variables, including total cholesterol (Total chol.), high-density lipoprotein cholesterol (HDL chol.), and triglycerides (TG). However, low-density lipoprotein cholesterol (LDL chol.) and fasting plasma glucose (FPG) did not differ significantly between females and males. As expected, cardiopulmonary exercise test variables showed significant sex differences. Males had higher values of oxygen consumption at peak (VO_2_peak) and at the ventilatory threshold (VO_2_thres), as well as higher oxygen consumption per unit of work (VO_2_/Work) and workload (WattPeak). Peak exercise haemodynamic responses also differed significantly between sexes, with males showing higher peak systolic blood pressure (SBPpeak), peak diastolic blood pressure (DBPpeak), and maximal rate–pressure product (RPPmax). No significant differences were observed in respiratory quotient (RQ), heart rate at the ventilatory threshold (HRthres), or maximal heart rate during cardiopulmonary exercise testing (HRpeak). Some autonomic indices, such as the RR interval (RR), the normalized unit power of the low-frequency component of RR variability (RR LFnu), the normalized unit power of the high-frequency component (RR HFnu), and their ratio (LF/HF), showed significant differences between female and male participants. However, the Autonomic Nervous System Index (ANSI) did not differ significantly between females and males, consistent with its design.

**Table 1:** Individual anthropometric, hemodynamic, biochemical, autonomic, and cardiopulmonary test parameters in all subjects, as well as in females and males.

		All	Females n = 103	Males n = 97	Mann–Whitney U Test
Variable	Unit	Mean ± SD	Mean ± SD	Mean ± SD	*p* Value
Age	[yrs]	37.48 ± 13.79	37.66 ± 12.73	37.29 ± 14.90	0.825
Smoke (No/Yes)	[%]	85.1/14.9	88.9/11.1	81.1/18.9	0.165
Weight	[kg]	74.28 ± 17.25	67.00 ± 16.77	82.01 ± 14.17	**<0.001**
Height	[cm]	169.63 ± 9.33	163.23 ± 6.51	176.41 ± 6.70	**<0.001**
WC	[cm]	90.32 ± 15.47	87.10 ± 15.70	93.76 ± 14.53	**0.002**
BMI	[kg/m^2^]	25.80 ± 5.54	25.18 ± 6.06	26.45 ± 4.88	**0.011**
SAP	[mmHg]	117.99 ± 13.23	114.65 ± 14.08	121.54 ± 11.29	**<0.001**
DAP	[mmHg]	72.17 ± 10.56	71.51 ± 10.85	72.87 ± 10.24	0.169
HR	[beats/min]	65.62 ± 9.60	67.10 ± 8.64	64.05 ± 10.33	**0.011**
FPG	[mg/dL]	89.00 ± 10.00	87.55 ± 9.98	92.03 ± 10.86	0.057
Total chol.	[mg/dL]	208.54 ± 39.96	216.09 ± 42.85	197.51 ± 32.69	**0.038**
HDL chol.	[mg/dL]	56.70 ± 15.09	62.56 ± 13.78	48.46 ± 11.76	**<0.001**
LDL chol.	[mg/dL]	127.38 ± 37.66	133.03 ± 38.85	117.49 ± 34.49	0.096
TG	[mg/dL]	106.00 ± 56.00	92.55 ± 37.04	124.72 ± 71.70	**0.041**
RR	[ms]	934.15 ± 139.22	909.03 ± 117.77	960.83 ± 155.06	**0.011**
RR TP	[ms^2^]	2958.65 ± 3122.38	2830.03 ± 2969.41	3095.23 ± 3286.99	**<0.001**
RR LFa	[ms^2^]	777.40 ± 792.61	678.29 ± 656.91	882.64 ± 906.66	0.100
RR HFa	[ms^2^]	1094.32 ± 1981.99	1146.80 ± 1977.46	1038.58 ± 1995.55	0.080
RR LFnu	[nu]	49.65 ± 20.65	43.49 ± 18.57	56.18 ± 20.83	**<0.001**
RR HFnu	[nu]	44.21 ± 19.85	49.41 ± 18.03	38.69 ± 20.28	**<0.001**
LF/HF	[ratio]	2.71 ± 5.59	1.22 ± 1.07	3.39 ± 7.81	**<0.001**
ΔLF	[nu]	29.52 ± 23.56	30.68 ± 22.80	28.29 ± 24.39	0.528
ANSI	[%]	57.35 ± 26.23	57.58 ± 26.02	57.10 ± 26.59	0.943
VO_2_rest	[mL·kg^−1^·min^−1^]	4.79 ± 2.35	4.31 ± 1.69	5.27 ± 2.79	**0.025**
VO_2_thres	[mL·kg^−1^·min^−1^]	18.07 ± 7.70	15.60 ± 5.14	20.61 ± 9.00	**<0.001**
VO_2_peak	[mL·kg^−1^·min^−1^]	31.62 ± 11.26	26.56 ± 8.65	36.99 ± 11.25	**<0.001**
VO_2_/Work	[mL·min^−1^·W^−1^]	10.15 ± 3.13	8.48 ± 2.49	11.85 ± 2.79	**<0.001**
HR/VO_2_	[beats·L^−1^·min]	5.67 ± 5.05	6.26 ± 4.66	5.10 ± 5.36	**<0.001**
VE/VCO_2_	[-]	25.27 ± 4.36	25.89 ± 4.36	24.62 ± 4.29	**0.035**
HRthres	[beats/min]	123.48 ± 18.97	125.01 ± 18.64	121.87 ± 19.28	0.185
HRpeak	[beats/min]	163.89 ± 16.09	163.30 ± 15.76	164.52 ± 16.49	0.619
SBPpeak	[mmHg]	157.61 ± 21.90	149.01 ± 21.61	163.81 ± 20.13	**<0.001**
DBPpeak	[mmHg]	79.92 ± 11.30	77.30 ± 10.90	81.90 ± 11.20	**0.008**
RPPmax	[beats·min^−1^·mmHg/100]	270.41 ± 43.62	258.60 ± 39.21	279.01 ± 44.80	**0.005**
RQ	[-]	1.16 ± 0.10	1.16 ± 0.10	1.17 ± 0.09	0.968
WattPeak	[W]	202.62 ± 46.72	171.50 ± 29.14	234.72 ± 39.14	**<0.001**

Abbreviations: Age = age (years); Smoke (No/Yes) = smoking status (current smoker: No/Yes); Weight = body weight (kg); Height = body height (cm); WC = waist circumference (cm); BMI = body mass index (kg/m^2^); SAP = systolic arterial pressure (mmHg); DAP = diastolic arterial pressure (mmHg); HR = heart rate (beats/min); FPG = fasting plasma glucose (mg/dL); Total chol. = total cholesterol (mg/dL); HDL chol. = high-density lipoprotein cholesterol (mg/dL); LDL chol. = low-density lipoprotein cholesterol (mg/dL); TG = triglycerides (mg/dL); RR = RR interval (ms); RR TP = total power of RR interval variability derived from the autoregressive spectrum of the tachogram (ms^2^); RR LFa = absolute power of the low-frequency spectral component of RR variability (ms^2^); RR HFa = absolute power of the high-frequency spectral component of RR variability (ms^2^); RR LFnu = normalized unit power of the low-frequency spectral component of RR variability; RR HFnu = normalized unit power of the high-frequency spectral component of RR variability; LF/HF = ratio between low- and high-frequency components of RR variability; ΔLF = difference between upright and resting low-frequency spectral component of RR variability in normalized unit; ANSI = Autonomic Nervous System Index (%); VO_2_rest = oxygen consumption at rest; VO_2_thres = oxygen consumption at ventilatory threshold; VO_2_peak = maximal oxygen consumption attained during cardiopulmonary test; VO_2_/Work = oxygen consumption per unit of work; HR/VO_2_ = heart rate to oxygen uptake ratio; VE/VCO_2_ = ventilatory equivalent for carbon dioxide; HRthres = heart rate at ventilatory threshold (beats/min); HRpeak = maximal heart rate attained during cardiopulmonary test (beats/min); SBPpeak = peak systolic blood pressure during CPX (mmHg); DBPpeak = peak diastolic blood pressure during CPX (mmHg); RPPmax = maximal rate–pressure product, calculated as peak systolic blood pressure × peak heart rate/100 (beats·min^−1^·mmHg/100); RQ = respiratory quotient; WattPeak = peak workload achieved during cardiopulmonary exercise testing (W). Data were collected when the total cholesterol cut-off for the normal range was set at 220 mg/dL. Data are presented as Mean ± SD; statistical comparisons refer to females versus males (*p* < 0.05). Values in bold indicate *p* < 0.05.

### 3.2. Correlation Analyses (Table 2 and Appendix A Table A1)

Table 2 summarizes the correlations of VO_2_peak and VO_2_peak percentile with the autonomic variables. ANSI showed a moderate positive correlation with both VO_2_peak (r = 0.418, *p* < 0.001) and VO_2_peak percentile (r = 0.457, 95% CI 0.340–0.560; *p* < 0.001). Although standardization of VO_2_peak for age and sex reduced the magnitude of correlations with most autonomic indices, ANSI still showed a moderate, statistically significant association. The complete correlation matrix, including anthropometric, haemodynamic, and secondary CPX variables, is provided in Appendix A Table A1.

**Table 2:** Correlations of VO_2_peak and VO_2_peak percentile with the autonomic variables.

	RR	RR TP	RR LFa	RR LFnu	RR HFa	RR HFnu	LF/HF	ΔLF	ANSI
VO_2_peak	**0.402 ‡**	**0.405 ‡**	**0.365 ‡**	−0.113	**0.308 ‡**	0.156 *	−0.105	**0.366 ‡**	**0.418 ‡**
VO_2_peak percentile	**0.406 ‡**	**0.214 †**	0.103	**−0.204 †**	0.145 *	**0.187 †**	−0.181 *	0.182 *	**0.457 ‡**

Abbreviations: VO_2_peak = maximal oxygen consumption attained during cardiopulmonary test; VO_2_peak percentile = percentile of maximal oxygen consumption attained during cardiopulmonary test derived from age- and sex-specific reference values; RR = RR interval; RR TP = total power of RR interval variability derived from the autoregressive spectrum of the tachogram; RR LFa = absolute power of the low-frequency spectral component of RR variability; RR LFnu = normalized unit power of the low-frequency spectral component of RR variability; RR HFa = absolute power of the high-frequency spectral component of RR variability; RR HFnu = normalized unit power of the high-frequency spectral component of RR variability; LF/HF = ratio between low- and high-frequency components of RR variability; ΔLF = difference between upright and resting low-frequency spectral component of RR variability in normalized unit; ANSI = Autonomic Nervous System Index. Sign = * *p* < 0.05; † *p* < 0.01; ‡ *p* < 0.001. Significant values are in bold.

### 3.3. Trend Analyses (Figure 1)

**Figure 1:**
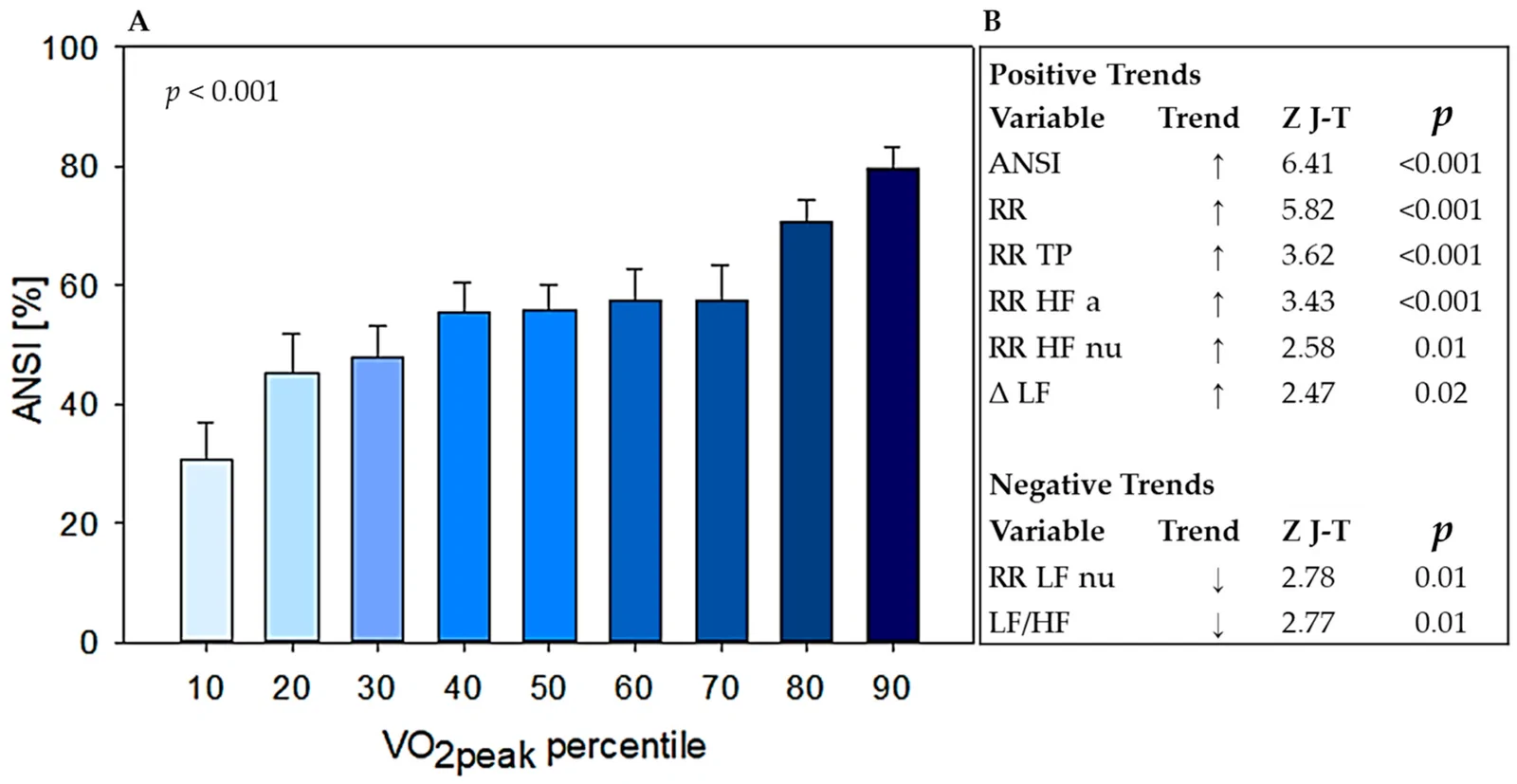
Trend in Autonomic Nervous System Index. Panel (**A**): ANSI mean values and standard error (SE) across nine predefined age- and sex-specific FRIEND VO2peak percentile levels [47]. Category sample sizes: 10th percentile, n = 15; 20th, n = 15; 30th, n = 24; 40th, n = 22; 50th, n = 34; 60th, n = 22; 70th, n = 17; 80th, n = 28; and 90th, n = 23. Panel (**B**): Jonckheere–Terpstra trend statistics. ANSI showed the largest Jonckheere–Terpstra trend statistic among the autonomic indices examined.

Across the nine VO_2_peak percentiles, Jonckheere–Terpstra tests detected significant trends in many autonomic indices. A positive trend was observed for ANSI (Figure 1), RR interval, RR TP, RR HFnu and ΔLF. Conversely, inverse trends were observed for RR LFnu and the LF/HF ratio. Among the indices considered, ANSI had the largest trend statistic.

### 3.4. Regression Analyses (Table 3)

Simple and multiple regression models were used to evaluate associations between autonomic indices and CRF, using both absolute VO_2_peak and VO_2_peak percentile as dependent variables (Table 3). In the simple analyses, ANSI showed the largest individual association among the autonomic variables considered. For absolute VO_2_peak, the simple ANSI model yielded R^2^ = 0.175 (Cohen’s f^2^ = 0.212), whereas the final multiple autonomic model explained 36.7% of the variance (R^2^ = 0.367; f^2^ = 0.580). VIF values ranged from 2.36 to 4.08, indicating no severe multicollinearity. For VO_2_peak percentile, the simple ANSI model explained 20.9% of the variance (R^2^ = 0.209; f^2^ = 0.264), with B = 0.426 (95% CI 0.310–0.542) and standardized β = 0.457 (*p* < 0.001). Importantly, in the model, ANSI was the only autonomic variable retained for VO_2_peak percentile.

Additional analyses were conducted to account for potential confounding. After adjustment for age, sex, smoking status, and BMI, ANSI was the only autonomic variable selected and remained independently associated with VO_2_peak percentile (β = 0.374, *p* < 0.001). The model including ANSI explained 45.6% of the variance (R^2^ = 0.456). A sensitivity analysis replacing BMI with WC yielded consistent findings: a comparable proportion of variance explained (R^2^ = 0.454), with ANSI again the only autonomic variable selected (β = 0.323, *p* < 0.001). The concordant pattern across different models confirmed the robustness of the association between ANSI and VO_2_peak percentile. Finally, ordinal regression provided a complementary assessment of the association between ANSI and the VO_2_peak percentile. In particular, each one-point increase in ANSI was associated with an odds ratio of 1.034 (95% CI 1.023–1.045; *p* < 0.001) for belonging to a higher VO_2_peak percentile category derived from the FRIEND registry. Expressed over a 10-point ANSI difference, this corresponds to an OR of 1.39 (95% CI 1.26–1.55).

**Table 3:** Simple and multiple regression models between autonomic variables and VO_2_peak (Panel (**A**)) or VO_2_peak percentile (Panel (**B**)).

**Panel (A)**			
**Regression Type**	**Significant Autonomic Variables Retained** **(*p* < 0.05)**	**Coefficient of** **Determination (R^2^)**	**Cohen’s f^2^**
Simple regression	RR	0.162	0.193
Simple regression	RR TP	0.164	0.196
Simple regression	RR HFnu	0.024	0.025
Simple regression	ΔLF	0.134	0.155
Simple regression	ANSI	0.175	0.212
Simple regression	RR LFa	0.133	0.153
Simple regression	RR HFa	0.095	0.105
Multiple regression	ANSI, RR LFa, ΔLF, RR, RR LFnu, RR TP	0.367	0.580
**Panel (B)**			
**Regression Type**	**Significant Autonomic Variables Retained** **(*p* < 0.05)**	**Coefficient of** **Determination (R^2^)**	**Cohen’s f^2^**
Simple regression	RR	0.165	0.198
Simple regression	RR TP	0.046	0.048
Simple regression	RR LFnu	0.042	0.044
Simple regression	RR HFnu	0.035	0.036
Simple regression	LF/HF	0.033	0.034
Simple regression	ΔLF	0.033	0.034
Simple regression	ANSI	0.209	0.264
Simple regression	RR HFa	0.021	0.021
Multiple regression	ANSI	0.209	0.264

Abbreviations: VO_2_peak = maximal oxygen consumption attained during cardiopulmonary test; VO_2_peak percentile = percentile of maximal oxygen consumption attained during cardiopulmonary test derived from age- and sex-specific reference values; RR LFa = absolute power of the low-frequency spectral component of RR variability (ms^2^); RR HFa = absolute power of the high-frequency spectral component of RR variability (ms^2^); RR LFnu = normalized unit power of the low-frequency spectral component of RR variability; RR HFnu = normalized unit power of the high-frequency spectral component of RR variability; LF/HF = ratio of low- to high-frequency components of RR variability; ΔLF = difference between upright and resting low-frequency spectral component of RR variability in normalized unit; ANSI = Autonomic Nervous System Index (%). Effect size according to Cohen’s [48] guidelines: f^2^ ≥ 0.02, f^2^ ≥ 0.15, and f^2^ ≥ 0.35 represent small, medium, and large effect sizes, respectively. Autonomic variables considered in both models as predictors: RR, RR TP, RR LFa, RR HFa, RR LFnu, RR HFnu, LF/HF, ΔLF, and ANSI.

## 4. Discussion

In this cross-sectional study, we found a significant association between the Autonomic Nervous System Index (ANSI)—a composite percentile-ranked index of cardiac autonomic regulation—and cardiorespiratory fitness (CRF), directly assessed by cardiopulmonary exercise testing (CPX). ANSI showed a moderate association with both VO_2_peak and age- and sex-standardized VO_2_peak percentile. Importantly, its association with VO_2_peak percentile remained significant after adjustment for age, sex, smoking status, and BMI and was confirmed in a parallel analysis replacing BMI with WC. When the autonomic variables were evaluated within the same adjusted framework, ANSI was the only autonomic variable selected. These findings support our hypothesis that an integrated, age- and sex-standardized autonomic index captures information meaningfully associated with directly measured CRF while also indicating that autonomic regulation represents only one component of the complex determinants of exercise capacity. It is widely recognized that exercise training induces cardiovascular and autonomic adaptations, including cardiac neural remodelling and enhanced vagal modulation [7,18,29,35,37,38,39], whereas autonomic dysregulation is commonly observed in several chronic conditions [18,21,22,24]. Spectral analysis of Heart Rate Variability (HRV) is a simple, non-invasive and cost-effective technique used to assess cardiac autonomic modulation, reflecting the interaction between sympathetic and vagal influences on the heart. It is now considered the “de facto” methodology for studying ANS control [49]. In this context, the use of HRV to study the association between cardiorespiratory fitness, a strong and consistent predictor of mortality [2,4,50], and ANS control is of particular importance. Nevertheless, the literature presents an inconsistent picture in this regard, due to a lack of consensus on methodologies and the interpretation of the metrics used to extract underlying information [41,42,43,44,45,46]. Importantly, several previous investigations relied on estimated rather than directly measured CRF. Grant et al. reported that heart rate explained a greater proportion of the variability in estimated VO_2_max than HRV measures [43], and Plaza-Florido et al. similarly found heart rate to be more strongly associated with CRF than HRV [44]. Conversely, Buchheit et al. reported associations between vagal-related HRV indices and CRF [41], Dostal et al. described an association between estimated CRF and RMSSD [42], Tekin et al. reported correlations between spectral HRV variables and maximal oxygen consumption in athletes [46], and Sala et al. observed associations between HRV-derived variables and ordered categories of aerobic fitness [45]. The current findings extend previous research by demonstrating an association between ANSI and directly measured VO_2_peak. In addition, we examined this relationship using age- and sex-standardized FRIEND VO_2_peak percentiles, thereby accounting for two major determinants of CRF [47].

A central aspect of the present study is the use of ANSI, rather than relying exclusively on individual HRV-derived indices. This index was developed to integrate information from different autonomic dimensions into a single composite percentile-ranked proxy of autonomic balance, with higher values indicating better autonomic control [33]. The 0–100 ranking facilitates simple comparisons among individuals, conditions or time periods and is readily understandable. Previous studies have applied ANSI in different exercise-related settings, including patients undergoing aerobic training [32], Olympic athletes characterized by different training loads and cardiac adaptations [51], and football players according to their playing positions [52]. The magnitude of the observed associations also warrants consideration. ANSI correlated moderately with VO_2_peak percentile (r = 0.457) and explained 20.9% of its variance in the simple regression model. Adjustment for major available confounders (age, sex, smoking status, and anthropometric measures) did not eliminate the association. The complementary ordinal analysis was consistent with these findings: each 10-point increase in ANSI was associated with approximately 40% higher odds of belonging to a higher CRF category (OR = 1.39). Taken together, these analyses indicate a consistent relationship between higher ANSI and higher CRF. At the same time, the moderate magnitude of the association makes clear that a substantial proportion of CRF variability is explained by factors beyond the autonomic variables considered in the present study. From a clinical perspective, autonomic evaluation is widely employed across a broad spectrum of settings [30,31], including exercise [40], sports medicine [53], and cardiovascular disease, in which autonomic dysfunction is an important pathophysiological mechanism and a potential therapeutic target [24,54]. Although the strength of the association between ANSI and CRF was not markedly greater than that observed for some other single HRV-derived variables, ANSI offers the advantage of integrating multiple aspects of cardiac autonomic regulation into a single composite index (ranging from 0 to 100, with higher values representing better autonomic function), reducing the interpretative complexity of considering several interrelated HRV indices separately. This characteristic may be particularly useful when autonomic assessment is performed in populations heterogeneous in age and sex. Accordingly, the possibility that ANSI might complement CPX in longitudinal follow-up or exercise-based programs, including circumstances in which repeated CPX is not feasible, should currently be regarded as a hypothesis requiring prospective studies.

### Limitations

This study has several limitations. First, its cross-sectional design does not allow causal or temporal relationships between cardiac autonomic regulation and CRF to be established. Accordingly, the observed association cannot demonstrate whether changes in ANSI parallel within-person changes in CRF, including those occurring in response to exercise training.

Second, although the additional multivariable analyses accounted for major available demographic, anthropometric, and behavioural covariates, including age, sex, smoking status, and either BMI or waist circumference, residual confounding remains possible. In particular, standardized information on habitual physical activity, including minutes of moderate-to-vigorous physical activity, was not available and could not be incorporated as an additional behavioural covariate. Other potentially relevant determinants of CRF, including haematological, immunological, endocrine, and additional clinical factors, were also not comprehensively available.

Third, biochemical measurements were available in only 89 participants because fasting blood sampling was not routinely performed at every preventive evaluation but according to the individual cardiovascular risk profile. Accordingly, these variables were treated as descriptive and not included in the principal regression analyses.

Fourth, the study population comprised a selected cohort (our population was relatively young and free from overt cardiometabolic disease) undergoing assessment in an Exercise Medicine setting, and several clinical conditions and treatments that could substantially affect autonomic regulation or exercise capacity were excluded. The present findings should therefore not be directly extrapolated to patients with established cardiovascular or metabolic diseases, individuals receiving medications that substantially influence autonomic function, or patients enrolled in cardiac or multidisciplinary rehabilitation programs. These populations may be particularly relevant to the potential clinical application of autonomic assessment but require dedicated validation.

Fifth, composite autonomic measures also have inherent limitations: they simplify the multidimensional nature of autonomic regulation but do not provide the same component-specific information as individual HRV measures [55]. Accordingly, the clinical utility of ANSI should be interpreted within the population and context in which it was evaluated [55]. In addition, because ANSI is a composite index derived from RR, RR TP, and ΔLF, some degree of correlation between ANSI and its individual components is inherent by design, although no severe multicollinearity was observed in the regression models. The use of a forward-selection procedure to build this index may represent a limitation since several of the autonomic variables examined are physiologically and statistically interrelated.

Finally, the present study was not designed to assess diagnostic agreement between ANSI and CPX, define clinically meaningful ANSI thresholds, evaluate responsiveness to longitudinal changes in CRF, or determine longitudinal predictive performance. Prospective studies with repeated ANSI and CPX assessments are required to determine whether within-person changes in ANSI parallel changes in CRF and whether ANSI provides clinically useful incremental information in broader cardiovascular, metabolic, and rehabilitation populations.

## 5. Conclusions

In this cross-sectional study, better cardiac autonomic regulation was consistently associated with higher CRF. Among the HRV indices examined, ANSI showed the strongest statistical association with CRF among the autonomic indices examined and was the only autonomic variable retained in the multiple regression model with VO_2_peak percentile. This association remained robust after adjustment for age, sex, smoking status, and anthropometric measures. These findings suggest that ANSI may capture the autonomic component associated with CRF more effectively than individual HRV indices while offering a simple tool for assessing CAR across individuals of different ages, sexes, and CRF levels. Prospective studies with repeated assessments of ANSI and CPX are needed to determine whether ANSI could track longitudinal changes in CRF and to assess its potential complementary role alongside CPX in the clinical assessment and follow-up of exercise-based interventions.

## Data Availability

The dataset used in this study is deposited in the Zenodo repository https://doi.org/10.5281/zenodo.18921671. Requests to access the dataset should be directed to daniela.lucini@unimi.it.

## Abbreviations

The following abbreviations are used in this manuscript:

Abbreviations	Full Title
ANS	Autonomic Nervous System
ANSI	Autonomic Nervous System Index
BMI	Body Mass Index
CAR	Cardiac Autonomic Regulation
CI	Confidence Interval
CPX	Cardiopulmonary Exercise Testing
CRF	Cardiorespiratory Fitness
DAP	Diastolic Arterial Pressure
DBPpeak	Peak Diastolic Blood Pressure during CPX
FPG	Fasting Plasma Glucose
FRIEND	Fitness Registry and the Importance of Exercise
HDL	High-Density Lipoprotein
HR	Heart Rate
HR/VO_2_	Heart Rate-to-Oxygen Uptake Ratio
HRpeak	Peak Heart Rate
HRthres	Heart Rate at Ventilatory Threshold
HRV	Heart Rate Variability
LDL	Low-Density Lipoprotein
LF/HF	Ratio of Low- to High-Frequency Components of RR Variability
N	Number
OR	Odds Ratio
PIN	Probability of F to Enter
POUT	Probability of F to Remove
R^2^	Coefficient of Determination
RMSSD	Root Mean Square of Successive Differences
RPPmax	Maximal Rate–Pressure Product
RQ	Respiratory Quotient
RR	RR Interval
RR HFa	Absolute High-Frequency Power of RR Variability
RR HFnu	Normalized High-Frequency Power of RR Variability
RR LFa	Absolute Low-Frequency Power of RR Variability
RR LFnu	Normalized Low-Frequency Power of RR Variability
RR TP	Total Power of RR Interval Variability
SAP	Systolic Arterial Pressure
SBPpeak	Peak Systolic Blood Pressure during CPX
SD	Standard Deviation
SE	Standard Error
TG	Triglycerides
VE/VCO_2_	Ventilatory Equivalent for Carbon Dioxide
VIF	Variance Inflation Factor
VO_2_max	Maximal Oxygen Uptake
VO_2_peak	Peak Oxygen Uptake
VO_2_rest	Oxygen Uptake at Rest
VO_2_thres	Oxygen Uptake at Ventilatory Threshold
VO_2_/Work	Oxygen Uptake per Unit of Work
WattPeak	Peak Workload
WC	Waist Circumference
ΔLF	Difference between Upright and Resting Normalized Low-Frequency Power of RR Variability

## Appendix A

**Table A1:** Correlation matrix of selected anthropometric, haemodynamic, autonomic and cardiopulmonary test variables.

	VO_2_peak	VO_2_peak perc.	VO_2_thres	VO_2_/Work	SAP	BMI	Waist	RR	RR TP	RR LFa	RR LFnu	RR HFa	RR HFnu	LF/HF	ΔLF	ANSI
VO_2_peak	–															
VO_2_peak perc.	**0.423 ‡**	–														
VO_2_thres	**0.797 ‡**	**0.369 ‡**	–													
VO_2_/Work	**0.684 ‡**	**0.234 †**	**0.597 ‡**	–												
SAP	−0.129	−0.068	−0.109	0.054	–											
BMI	**−0.519 ‡**	**−0.407 ‡**	**−0.403 ‡**	−0.118	**0.403 ‡**	–										
WC	**−0.480 ‡**	**−0.471 ‡**	**−0.418 ‡**	−0.106	**0.472 ‡**	**0.894 ‡**	–									
RR	**0.402 ‡**	**0.406 ‡**	**0.269 ‡**	**0.223 †**	−0.06	**−0.148 ***	**−0.172 ***	–								
RR TP	**0.405 ‡**	**0.214 †**	**0.322 ‡**	**0.259 ‡**	−0.097	**−0.234 ‡**	**−0.264 ‡**	**0.449 ‡**	–							
RR LFa	**0.365 ‡**	0.103	**0.336 ‡**	**0.238 †**	−0.068	**−0.145 ***	−0.144	**0.256 ‡**	**0.647 ‡**	–						
RR LFnu	−0.113	**−0.204 †**	−0.081	0.047	0.136	**0.250 ‡**	**0.313 ‡**	**−0.310 ‡**	**−0.369 ‡**	0.134	–					
RR HFa	**0.308 ‡**	**0.145 ***	**0.237 †**	**0.180 ***	−0.136	**−0.214 †**	**−0.260 ‡**	**0.348 ‡**	**0.903 ‡**	**0.442 ‡**	**−0.515 ‡**	–				
RR HFnu	**0.156 ***	**0.187 †**	0.111	−0.023	**−0.184 †**	**−0.276 ‡**	**−0.320 ‡**	**0.374 ‡**	**0.407 ‡**	−0.065	**−0.950 ‡**	**0.542 ‡**	–			
LF/HF	−0.105	**−0.181 ***	−0.108	−0.033	0.023	**0.224 †**	**0.226 †**	**−0.224 †**	**−0.147 ***	−0.022	**0.470 ‡**	**−0.155 ***	**−0.460 ‡**	–		
ΔLF	**0.366 ‡**	**0.182 ***	**0.275 ‡**	**0.179 ***	**−0.221 †**	**−0.352 ‡**	**−0.404 ‡**	**0.282 ‡**	**0.284 †**	0.003	**−0.681 ‡**	**0.346 ‡**	**0.644 ‡**	**−0.322 ‡**	–	
ANSI	**0.418 ‡**	**0.457 ‡**	**0.326 ‡**	**0.211 †**	**−0.185 †**	**−0.328 ‡**	**−0.396 ‡**	**0.730 ‡**	**0.577 ‡**	**0.328 ‡**	**−0.521 ‡**	**0.466 ‡**	**0.554 ‡**	**−0.312 ‡**	**0.606 ‡**	–

Abbreviations: VO_2_peak = maximal oxygen consumption attained during cardiopulmonary test; VO_2_peak perc. = percentile of maximal oxygen consumption attained during cardiopulmonary test derived from age- and sex-specific reference values; VO_2_thres = oxygen consumption at ventilatory threshold; VO_2_/Work = oxygen consumption per unit of work; SAP = systolic arterial pressure; BMI = body mass index; WC = waist circumference; RR = RR interval; RR TP = total power of RR interval variability derived from the autoregressive spectrum of the tachogram; RR LFa = absolute power of the low-frequency spectral component of RR variability; RR LFnu = normalized unit power of the low-frequency spectral component of RR variability; RR HFa = absolute power of the high-frequency spectral component of RR variability; RR HFnu = normalized unit power of the high-frequency spectral component of RR variability; LF/HF = ratio between low- and high-frequency components of RR variability; ΔLF = difference between upright and resting low-frequency spectral component of RR variability in normalized unit; ANSI = Autonomic Nervous System Index. Sign = * *p* < 0.05; † *p* < 0.01; ‡ *p* < 0.001. Significant values are in bold.
